# California wildfire spread derived using VIIRS satellite observations and an object-based tracking system

**DOI:** 10.1038/s41597-022-01343-0

**Published:** 2022-05-30

**Authors:** Yang Chen, Stijn Hantson, Niels Andela, Shane R. Coffield, Casey A. Graff, Douglas C. Morton, Lesley E. Ott, Efi Foufoula-Georgiou, Padhraic Smyth, Michael L. Goulden, James T. Randerson

**Affiliations:** 1 Department of Earth System Science, University of California, Irvine, CA USA; 2 Earth System Science Program, Faculty of Natural Sciences, Universidad del Rosario, Bogota, Colombia; 3 School of Earth and Environmental Sciences, Cardiff University, Cardiff, UK; 4 Department of Computer Science, University of California, Irvine, CA USA; 5 Biospheric Sciences Laboratory, NASA Goddard Space Flight Center, Greenbelt, MD USA; 6 Global Modeling and Assimilation Office, NASA Goddard Space Flight Center, Greenbelt, MD USA; 7 Department of Civil and Environmental Engineering, University of California, Irvine, CA USA

**Keywords:** Natural hazards, Fire ecology

## Abstract

Changing wildfire regimes in the western US and other fire-prone regions pose considerable risks to human health and ecosystem function. However, our understanding of wildfire behavior is still limited by a lack of data products that systematically quantify fire spread, behavior and impacts. Here we develop a novel object-based system for tracking the progression of individual fires using 375 m Visible Infrared Imaging Radiometer Suite active fire detections. At each half-daily time step, fire pixels are clustered according to their spatial proximity, and are either appended to an existing active fire object or are assigned to a new object. This automatic system allows us to update the attributes of each fire event, delineate the fire perimeter, and identify the active fire front shortly after satellite data acquisition. Using this system, we mapped the history of California fires during 2012–2020. Our approach and data stream may be useful for calibration and evaluation of fire spread models, estimation of near-real-time wildfire emissions, and as means for prescribing initial conditions in fire forecast models.

## Abstract (summary)

**Table:** 

Measurement(s)	Wildfire half-daily perimeters and attributes
Technology Type(s)	Remote sensing
Sample Characteristic - Organism	Wildfires
Sample Characteristic - Environment	Ecosystems
Sample Characteristic - Location	California

## Background & Summary

Fire is an integral process within the Earth system that influences ecosystem structure and atmospheric composition^1^. Climate warming, land management, and demographic trends have changed the role of fire in recent decades^2^, leading to new extremes in fire behavior that have created unprecedented environmental, societal, and climate impacts^3,4^. The distribution of fire sizes and day-to-day variations in fire behavior and fire spread rate are influenced by interactions between multiple weather and ecosystem processes, and it is still a challenge for current models to represent these complex physical and ecological processes correctly^5^. New observations are needed to characterize fire regimes (e.g., frequency, intensity, and severity) over different periods and regions, evaluate the impacts of fires on air quality, climate and ecosystems, and forecast fire occurrence and spread. While fire spread models, either with physics-based algorithms^6,7^ or empirical formulations^8–10^, are widely used to project the behavior of individual fires and regional ensembles, the evaluation of their performance is often limited by a lack of high-quality observations^11^.

Fire perimeter and area data were historically derived from field and aerial observations. Since the late 1970s, satellite remote sensing instruments, particularly from Landsat, have provided an alternative, reliable data source for fire area and fire severity mapping^12^. In many early applications of satellite datasets, fire detections were often reported as a series of independent, pixel-level events on a spatial grid, which often ignored the spatial and temporal linkages between them. Recent studies have used ideas from object-oriented classification and contextual growth to track the properties of individual fires using pixel-level fire data^13–19^ (Table 1). By clustering fire pixels that are detected nearby in time and space, this type of approach maximizes the benefit of routine satellite observations of fires. The most widely used data in these studies are burned area products from mid-resolution infrared imaging sensors (*e.g*., Moderate Resolution Imaging Spectroradiometer, MODIS). However, these products are often not suitable for generating a rapid assessment of fire events. This is because a sustained interval of post-fire surface reflectance observations is needed in the change detection algorithm used for estimating burned area. Alternatively, the thermal anomaly and radiative power detected by satellites provide instantaneous information on the location and energy release of active fires^20^, which allows for the detection of small fires and delineation of fire events in near-real-time^16,21,22^. However, the spatiotemporal coverage of active fire detections is incomplete as a consequence of fire spread (and sometimes fire extinction) in between consecutive satellite overpass times, and as a consequence of land surface masking by clouds and fire aerosols^23^.

**Table 1:** A list of recent studies delineating fire events using satellite fire observations.

Study	Region	Time period	Satellite fire detection	Spa. res.	Temp.res.	Geospatial approach (spatial, temporal)	NRT product	External perimeter required	Vector output	Fire size	Product
*Lobeoda and Csiszar, 2007*^35^	Northern Eurasia	2001–2009	MODIS AF (MOD14)	1 km	Daily	Spatiotemporal (2.5 km, 4-day)	No	No	No	All	—
*Archibald and Roy, 2009*^19^	Southern Africa	2000-2008	MODIS BA (MCD45)	500 m	Daily	Spatiotemporal (touched, 8-day)	No	No	No	All	—
*Archibald et al., 2013*^36^	Global	1997–2010	MODIS BA (MCD45)	500 m	Daily	Spatiotemporal (touched, 2-day)	No	No	No	All	—
*Balch et al., 2013*^37^	The Great Basin, USA	2000–2009	MODIS BA (MCD45)	500 m	Daily	Spatiotemporal (touched, 2-day)	No	No	No	All	—
*Veraverbeke et al., 2014*^38^	Alaska and western US	2007–2012	MODIS BA (MCD45, MCD64), AF (MCD14)	500 m 1 km	Daily	Kriging model	No	Yes	No	Selected large fires	—
*Loepfe et al., 2014*^39^	Europe	2001–2010	MODIS AF (MOD 14)	1 km	Daily	Propagation algorithm (11 km, 1-day)	No	No	No	> 2 hot spots	—
*Hantson et al., 2015*^40^	Global	2002–2010	MODIS BA (MCD45)	500 m	Daily	Spatiotemporal (touched, 14-day)	No	No	No	All	—
*Oom et al., 2016*^41^	Global	2003	MODIS BA (MCD45)	500 m	Daily	Spatiotemporal (touched, 2-day, 8-day, 14-day)	No	No	No	All	—
*Frantz et al., 2016*^42^	Southern Africa	2010	MODIS BA (MCD45)	500 m	Daily	Spatiotemporal (touched, 1-day, 3-day, 5-day)	No	No	No	All	—
*Benali et al., 2016*^43^	5 regional zones	2000–2013	MODIS AF (MCD14)	1 km	Daily	Temporally constrained clustering algorithm	No	Yes	No	All	—
*Nogueira et al., 2017*^44^	Brazil savannas	2002–2009	MODIS BA (MCD45) and Fire-CCI BA	500 m	Daily	Spatiotemporal (touched, 8-day)	No	No	Yes (fitted ellipses)	All	—
*Laurent et al., 2018*^18^	Global	2005–2011	MODIS BA (MCD64) and MERIS BA	500 m 300 m	Daily	Spatiotemporal (touched, 3-day, 5-day, 9-day, 14-day)	No	No	Yes (fitted ellipses)	> 5 burned pixels	FRY
*Artes et al., 2019*^14^	Global	2000–2018	MODIS BA (MCD64)	500 m	Daily	Spatiotemporal (touched, 5-day, 16-day)	No	No	No	All	GlobFire
*Andela et al., 2019*^13^	Global	2003–2016	MODIS BA (MCD64)	500 m	Daily	Local minima and fire persistence thresholds	No	No	No	All	Fire Atlas
*Balch et al., 2020*^17^	CONUS	2001–2019	MODIS BA (MCD64)	500 m	Daily	Spatiotemporal (2315 m, 11-day)	No	No	Yes	All	FIRED
*Scaduto et al., 2020*^21^	Northern California	2017–2018	MODIS AF (MCD14) and VIIRS AF (I-band)	500 m 375 m	Daily	Three geospatial interpolation approaches	Yes	Yes	No	> 5000 acres	—
*Lizundia-Loiola et al., 2020*^16^	Global	2001–2018	MODIS AF (MCD14)	1 km	Daily	Spatiotemporal (1875 m, 4-day)	No	No	No	All	—
*This study*	California	2012–2020	VIIRS AF (I-band)	375 m	Half-daily	Progressive spatiotemporal aggregation (LCT dependent spatial thresholds, 5-day)	Yes	No	Yes	All	FEDS

AF and BA are acronyms of ‘Active Fire’ and ‘Burned Area’, respectively. The ‘Geospatial approach’ column includes spatial and temporal thresholds (shown within the parentheses) used for clustering fire pixels.

**Fig. 1:**
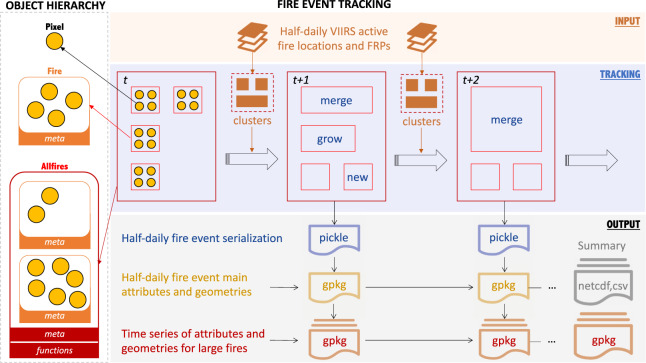
A conceptual diagram of the fire object tracking system. For each half-daily time step (t), new active fire detections from VIIRS (locations and fire radiative power, FRP) are used as inputs to modify the *pixel*, *fire* and *allfires* objects. Subsequently the properties and geometries of every *fire* object are dynamically tracked across time and space, and the output data are saved in 4 layers of products (shown in different colors) with different data formats (*Pickle*, *GeoPackage*, *NetCDF*, and CSV, see Table 4 for detail).

**Fig. 2:**
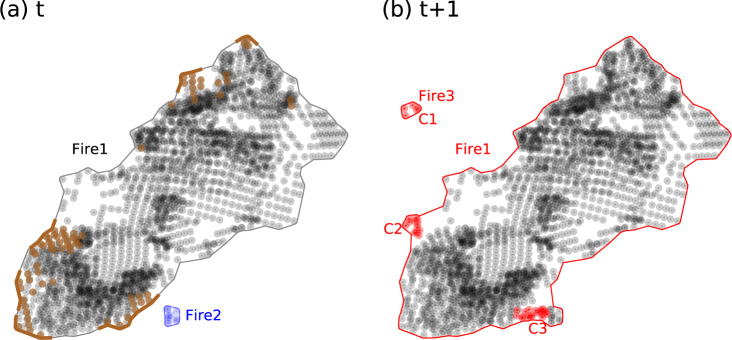
A schematic diagram of fire tracking. (**a**) Two idealized *fire* objects (Fire 1, Fire 2) at the time step *t*. Colored and grey dots represent newly detected and previously detected VIIRS active fire pixels. The brown segments along the fire perimeter are the active fire fronts. (**b**) At the next time step t + 1, new fire pixels (in red) are clustered (C1, C2, C3), and are used for forming a new *fire* object (C1 for Fire 3), or for the growth of an existing *fire* object (C2 for Fire 1). Expanded fire objects (Fire 2) are merged with an existing active *fire* object (Fire 1) if they grow close enough (due to the addition of C3). Vector shapes of the fire perimeter and active fire fronts for each *fire* object are updated at each time step.

Here we developed an automatic system (Fig. 1) to dynamically track the growth of all fire events at a regional scale, using active fire pixels recorded by the Visible Infrared Imaging Radiometer Suite (VIIRS) instrument^24^. For each 12-hour overpass, the VIIRS active fire pixels are extracted and grouped into different fire objects (Fig. 2). Fire attributes and vector shapes of the fire perimeter and active fire front line are recorded in the system at each time step. An alpha shape algorithm delineates the fire perimeter and fills in the gaps between the active fire detections in consecutive time steps^22^. This makes it possible to dynamically track changes in shape, active front, and other properties for each fire in a region in near-real-time.

By applying this algorithm to VIIRS active fire detections in California, we created an object-based Fire Events Data Suite (FEDS) over the past 9 fire seasons (2012–2020). The FEDS contains direct serialization of all fire objects, core fire properties, and vector geometries at each time step, the time series of attributes for large fires, and year-end summary files for wildfire statistics.

The system and the dataset presented here may be of potential interest to different stakeholders and research communities. FEDS provides a basis for analyzing the response of wildfires to variability in climate and land surface properties and for evaluating the performance of fire spread models. Specifically, the cumulative fire spread (over a 12-hour interval) from FEDS may inform optimization of model parameters regulating fire spread rates as a function of surface environmental conditions. It may also allow for more comprehensive comparisons across all large fires within a specific ecoregion or biome. In addition, dynamical tracking of the spatiotemporal evolution of fire perimeters provides a means (in future work) for the estimation of high-resolution fire emissions in near-real-time. Furthermore, this dataset can also potentially be used to support the development of machine learning methods that can improve the real-time prediction of the future evolution of active fires. This will be particularly important for risk evaluation, fire management, and air quality forecasts.

## Methods

### Overview

In this study, we used VIIRS active fire detections to track the dynamic evolution of all fires in California from 2012 to 2020 (Fig. 1). We developed an approach that has the following steps. First, after reading the satellite fire pixel data at each 12-hour time step, the new fire pixels are aggregated into multiple clusters using the fire pixel locations and an automatic clustering algorithm. These clusters are then spatially compared to existing fire objects. If a cluster is not close to any existing active fire object, we use all fire pixels within the cluster to form a new fire object. If a cluster is located near an existing fire object which is still active, we view the cluster as an extension of the existing fire. In this case, we append all pixels within the cluster to the corresponding existing fire object, allowing the existing object to grow. When a fire expands and gets close enough (within a pre-defined distance threshold) to an existing active fire object, we merge the two objects. For each time step (12 hours in this case for the two overpasses), we derive or update a suite of attributes and status indicators associated with each fire event, including pixel-level attributes of fire and surface properties, vector geometries related to the fire shape, and meta-attributes characterizing the entire fire object.

### Data input

Satellite remote sensing instruments provide active fire detections with accurate geographical location and broad spatial coverage. The primary data for this fire tracking system are active fire locations and the fire radiative power (FRP) recorded by the VIIRS instrument aboard the Suomi National Polar-orbiting Partnership (Suomi-NPP) satellite^24^. VIIRS observes Earth’s surface twice each day in low and mid latitude regions, with local overpass times of approximately 1:30 am and 1:30 pm. Compared to its predecessor, the MODIS sensors on the Terra and Aqua satellites, VIIRS has a higher spatial resolution and can detect smaller and cooler fires^24^. Also, the VIIRS instrument provides a more consistent pixel area across the image swath^25^, resulting in more accurate estimates of active fire location. Therefore, compared with MODIS, the VIIRS active fire products can be used to map fire event progression with higher accuracy^21^. Two streams of VIIRS active fire data are operationally produced using a contextual fire detection algorithm^24^, drawing upon VIIRS moderate resolution band (M-band) and imaging band (I-band) reflectance and radiance data layers. In this fire tracking system, we used the Suomi-NPP VIIRS I-band fire location data product (VNP14IMGML, Collection 1 Version 4) that contains the centre location, FRP, scan angle, and other attribute fields associated with each pixel. The I-band fire detection product has a 375-m spatial resolution at nadir (the sub-satellite point) and an average resolution across the full swath of about 470 m. Theoretical estimates of fire detection efficiency for the VIIRS sensor indicate that during the day, VIIRS can detect 700 K fires with 50% probability that have a size of about 200 m^2^ (a 15 m × 15 m fire area)^24^. During night, the detection efficiency increases, and VIIRS can detect 700 K fires as small as 40 m^2^. From a fire spread tracking perspective, these detection efficiencies imply that in many instances, the area of a fire pixel that is covered with flaming fire combustion is several orders of magnitude smaller than the overall pixel size. The VNP14IMGML data, available from 2012 onwards, were downloaded from the University of Maryland VIIRS Active Fire website (https://viirsfire.geog.umd.edu/).

Land cover data are an additional input in the system required to classify different fire types and determine the spatial connectivity threshold. Here we use the U.S. National Land Cover Database (NLCD 2016)^26^ that is available from the Multi-Resolution Land Characteristics (MRLC) Consortium website (https://www.mrlc.gov/national-land-cover-database-nlcd-2016). We aggregated the original 30-m data to match the spatial resolution of VIIRS active fire data, and merged the original 16 classes into several groups: ‘Water’, ‘Urban’, ‘Barren’, ‘Forest’, ‘Shrub’, ‘Grassland’, and ‘Agriculture’. We used the 1000-hour dead fuel moisture from the high-resolution (4 km) gridMET product^27^ for the purpose of separating wildfires and management fires. This gridMET dataset was computed from 7–day average conditions composed of day length, hours of rain, and daily temperature and humidity ranges. Regularly updated gridMET data are available from the Climatology Lab website (http://www.climatologylab.org/gridmet.html).

Other ancillary and validation datasets used in this study included a shapefile of California borders and fire perimeters from the California Forestry and Fire Protection’s Fire and Resource Assessment Program (FRAP) dataset (https://frap.fire.ca.gov/mapping/maps/).

### Fire object hierarchy

Fire detections from VIIRS are dynamically tracked within the framework of a three-level object hierarchy (Fig. 1). The lowest level is the fire *pixel* object, which includes the geographical location (latitude and longitude), the FRP value, and the origin (first assigned *fire* object *id*). The second level is the *fire* object, which includes all attributes associated with each individual fire event at a particular time step (Table 2). Each *fire* object includes one or more fire *pixel* objects, a unique identification number (*id*), and a set of attributes associated with the whole fire. Two types of fire attributes are derived and recorded for each *fire* object. The first type encompasses temporal (*e.g*., ignition time, duration) and spatial (*e.g*., centroid, ignition location) characteristics of the object as well as general properties (*e.g*., size, type, active status). The second type is the geometric information related to the *fire* object, including the fire perimeter, the active fire front line, and the newly detected fire pixel locations (stored as vectors). All *fire* objects in the State of California are combined to form an *allfires* object, to characterize the whole-region fire situation at a specific time step. The *allfires* object comprises a list of *fire* objects, and also contains meta information representing the statistics of all fires and the records describing fire evolution. A full list of the attributes associated with the *pixel* object, the *fire* object, and the *allfires* object is presented in Table 2.

**Table 2:** List of main attributes associated with *pixel*, *fire* and *allfires* objects.

OBJECT	TYPE	VARIABLE	MEANING	APPROACH
**Pixel**	Property	t	Time of detection	From VIIRS active fire pixel recording time
		FRP	Fire radiative power	From VIIRS active fire data
		origin	Fire *id* of originally assigned *fire* object	Recorded at time of creating a new fire object using the pixels
	Geometry	loc	Latitude and longitude	From VIIRS active fire location data
**Fire**	List of *pixel* objects	pixels	All pixel objects	Dynamically tracked using VIIRS active fire data
		newpixels	All pixel objects newly detected	Dynamically tracked using VIIRS active fire data
		ignpixels	All pixel objects formed at the time step of ignition	Recorded at the ignition time of the *fire* object
		flinepixels	All new pixel objects near the fire line	Determined from newpixels and fline
		extpixels	All pixel objects near the fire perimeter	Determined from pixels and hull
	Property	id	Fire identification number	Set at the time of object creation
		t_st, t_ed	First and last time when fire pixels are recorded	Recorded at the first and the last time steps when fire pixels associated with the fire object are detected
		duration	Duration of the fire	Calculated from t_st and t_ed
		invalid	Indicator of the validity of a *fire* object	Set to True if the fire object is merged to another object or classified as static fire
		isactive	The active status	Set to True if t_inactive < = 5 days
		t_inactive	Time length of inactive	Determined using t_ed and current time
		farea	Spatial area of the fire	Calculated from hull
		centroid	Centroid of the fire	Calculated from hull
		pixden	Fire pixel density	Calculated from pixel number and farea
		meanFRP	Mean FRP of the new fire pixels	Calculated from newpixels
		LCTmax	Dominant land cover type	Read from the NLCD data
		stFM1000	1000-hr fuel moisture at t_st	Read from the gridMET data
		ftype	Fire type	Determined using LCTmax and stFM1000
		fperim	Length of fire hull perimeter	Calculated from hull
		flinelen	Length of the active fire line	Calculated from fline
	Geometry	hull	Vector shape of fire perimeter	Derived by applying the alpha shape algorithm to all fire pixel locations
		fline	Vector shape of active fire line	Calculated from hull and new fire pixel locations
**Allfires**	List	fires	All fire objects	A collection of all fires at a time step
	Property	t	Current time step	Recorded in the model
		heritages	Fire merging history	Recorded when merging incidence occurs
		fids_expanded fids_new fids_merged fids_terminated	List of ids for fire objects with shape changes (expansion, forming, merging, termination)	Recorded when fire object changes occur

### Fire event tracking

The fire records (locations and FRPs) from the monthly VIIRS active fire location products (VNP14IMGML) are read into the system at each half-daily time step (roughly 1:30 am and 1:30 pm local time). We apply spatial and temporal filters to the data to extract active fire pixels recorded in California during each 12-hour time interval. We also apply quality flag filters (thermal anomaly type of ‘*0: presumed vegetation fire*’ in VNP14IMGML)) to ensure the use of only pixels likely associated with vegetation fires. The fire location and FRP values are used to create fire *pixel* objects. To speed up the calculation, the newly detected active fire pixels after filtering are first aggregated to specific clusters using the distances between them and an automatic clustering algorithm. In this initial aggregation algorithm, a ball tree^28^ is created to partition all newly detected active fire pixels into a nested set of hyperspheres in a 2-D space (latitude and longitude). This space partitioning data structure can be used to expedite nearest neighbours search^29^ and allow for quick cluster grouping. Here we refer to a cluster as a collection of *pixel* objects that are recorded at the same time step and are also spatially nearby. In the following steps, all pixels within a cluster are considered as a whole for fire merging and creation.

We define an extended area for every existing *fire* object as the fire vector perimeter (see the section of *Calculating and recording fire attributes* for detail) plus a radial buffer that depends on the fire type property of the object. The buffer is set to 5 km for forest fires and 1 km for other fire types (shrub, crop, urban), considering that the fire spread rate can differ across biomes^13^. We then evaluate the spatial distance between the perimeters of a newly classified cluster and all existing active *fire* objects (a *fire* object keeps an active status if one or more active fire pixels associated with it are detected during the past 5 days), and calculate the shortest distance. If the shortest distance is smaller than the buffer of the associated existing *active* fire (*i.e*., new cluster overlaps with the extended area of an existing *fire* object), we assume all fire pixels in the new cluster are associated with the growth of the existing *fire* object at the current time step (Fig. 2). The existing *fire* object is updated by appending all fire *pixel* objects within the new cluster. If a newly classified cluster does not overlap with the extended area of any existing active fire object, we assume this is a new fire. A new *fire* object (by assigning a new fire *id*) is created using all fire *pixel* objects in the cluster.

**Fig. 3:**
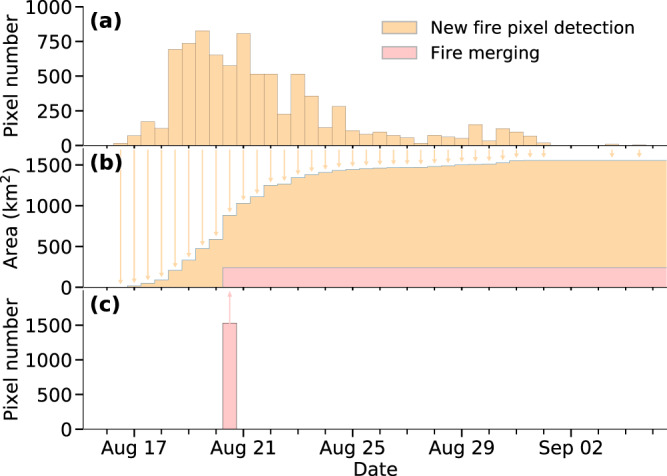
The time series of growth for the SCU Lightning Complex fire (2020). Panel (**b**) shows the fire size of the SCU fire (total area within the fire object perimeter) at half-daily time steps. A fraction of the fire growth (shown in orange) was due to the addition of newly detected fire pixels. Panel (**a**) shows the number of new fire pixels (associated with the SCU fire object) detected at each time step. The other part of the fire growth (shown in red) was due to the merging with existing fire objects. Panel (**c**) shows the number of fire pixels in the existing objects that were merged to the SCU fire object.

With the addition of new fire pixels, an existing *fire* object may expand and touch the extended area of another existing active *fire* object. If this happens, we assume that these two existing *fire* objects merge into a single object at this time step. All fire pixels in the *fire* object with a higher *id* number (a later start date, termed as the ‘*source fire*’) are appended to the *fire* object with lower *id* number (earlier start date, termed as the ‘*target fire*’) in this case. We record the *id* of the target fire in a list of fire mergers, and update all attributes associated with this fire (Fig. 3). In order to avoid double counting, the source *fire* object (with all pixels being transferred to the target *fire* object) is flagged as *invalid*, and is excluded from statistical analysis of fire events.

### Calculating and recording fire attributes

Other than individual fire pixels contained in a *fire* object, several core attributes (properties and geometries) are also dynamically updated at each time step and are used for fire tracking and characterization.

Important time-related attributes include the fire ignition time (the time step at which the first fire pixel within the fire object was detected), the fire end time (the latest time step with an active fire observation), and the fire duration (the time difference between the ignition time and end time). If a *fire* object does not have new active fire pixels appended during 5 consecutive days (*i.e*., the fire end time is more than 5 days before the present time step), its status is set to *inactive*. Once inactive, a *fire* object is no longer evaluated for use in future clustering (*i.e*., new active fire detections later will form new *fire* objects, even if they are spatially close to the inactive *fire* object).

Each *fire* object is assigned to a specific fire type. The fire type is identified using the major land cover type within the fire perimeter (Table 3). In an initial analysis, we found that prescribed fires, on average, have higher coarse fuel moisture levels than wildfires. Therefore, we also record the 1000-hour fuel moisture (fm1000) from the gridMET dataset^27^ for each fire object (corresponding to the ignition time step) and use this value to divide forest and shrub fires further to wildfire and prescribed types.

**Table 3:** Classification of fire types based on dominant land cover type (from the US National Land Cover Database) within each fire perimeter and the 1000-hr fuel moisture (FM-1000, from gridMET dataset) at the time of ignition.

DOMINANT LAND COVER TYPE	FM-1000	FIRE TYPE
**Forest**	> = 12%	Forest wildfire
	< 12%	Forest management fire
**Shrub, Grassland**	> = 12%	Shrub wildfire
	< 12%	Shrub management fire
**Agriculture**	N/A	Agricultural fire
**Urban**	N/A	Urban fire
**Water, Barren**	N/A	Other fire

An essential step in this object-based fire tracking system is to determine the vector shape of the fire perimeter. In this system, we use an alpha shape^30^ algorithm to derive bounding polygons containing fire pixels in a fire object. For an alpha shape, the radius of the disks forming the curves in the polygon is determined by the alpha parameter α. Compared with the commonly used convex hull, the alpha shape hull is able to capture the irregular shapes around the fire perimeter more accurately^22^.

**Fig. 4:**
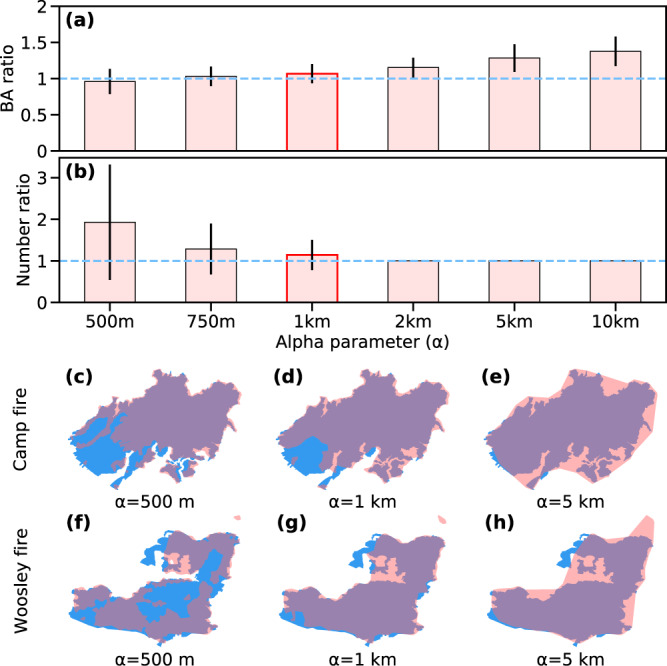
Optimization of the alpha shape parameter (α). For all large fires (final size > 4 km^2^) in California during 2018, fire perimeters were estimated using VIIRS active fires and different alpha parameters. By comparing (**a**) the burned area (BA) and (**b**) the number of fire objects with the FRAP data, an optimal alpha parameter of 1 km was identified for use in this study (shown in red). The vertical bars and lines show the mean and 1-std variability from all fires. The dashed blue lines indicate the ideal values when compared to FRAP. Panels (**c**)–(**h**) show the fire perimeters derived using different alpha shape parameters for two sample fires in 2018. The shapes with pink color are final FEDS fire perimeters derived from VIIRS active fires using the alpha shape algorithm. The blue shapes represent the corresponding fire perimeters from the FRAP dataset. Overlap between FRAP and FEDS is shown in purple.

To identify the optimal values for the α parameter, we performed the following analysis. First, we derived the final fire perimeters for all large fires that occurred in California during the 2018 wildfire season using a set of α values ranging from 500 m to 10 km and compared the results with more refined fire perimeters from the Fire and Resource Assessment Program (FRAP) dataset (Fig. 4). Large magnitude α values tended to overestimate the total burned area, while small α values often fragmented a large *fire* event. We found that a value of α = 1 km was optimal in terms of balancing the ability of the hull to catch the boundary shape and to keep the integrity of a *fire* object. For each time step, we applied the alpha shape algorithm to all fire pixel locations associated with a *fire* object since the time of ignition. This processing step resulted in a concave hull with the shape of polygon or multipolygon. To account for the pixel size, we expanded the concave hull to the fire perimeter using a buffer size equal to half of the VIIRS nadir cross-track pixel width (187.5 m). The alpha shape algorithm does not work when the total number of fire pixels (*npix*) is less than 4. If *npix* equals 3, we used a convex hull algorithm and the same 187.5 m buffer to determine a polygon perimeter. If *npix* is 1 or 2, circles centered on the fire pixel location with radius of 187.5 m were used.

**Fig. 5:**
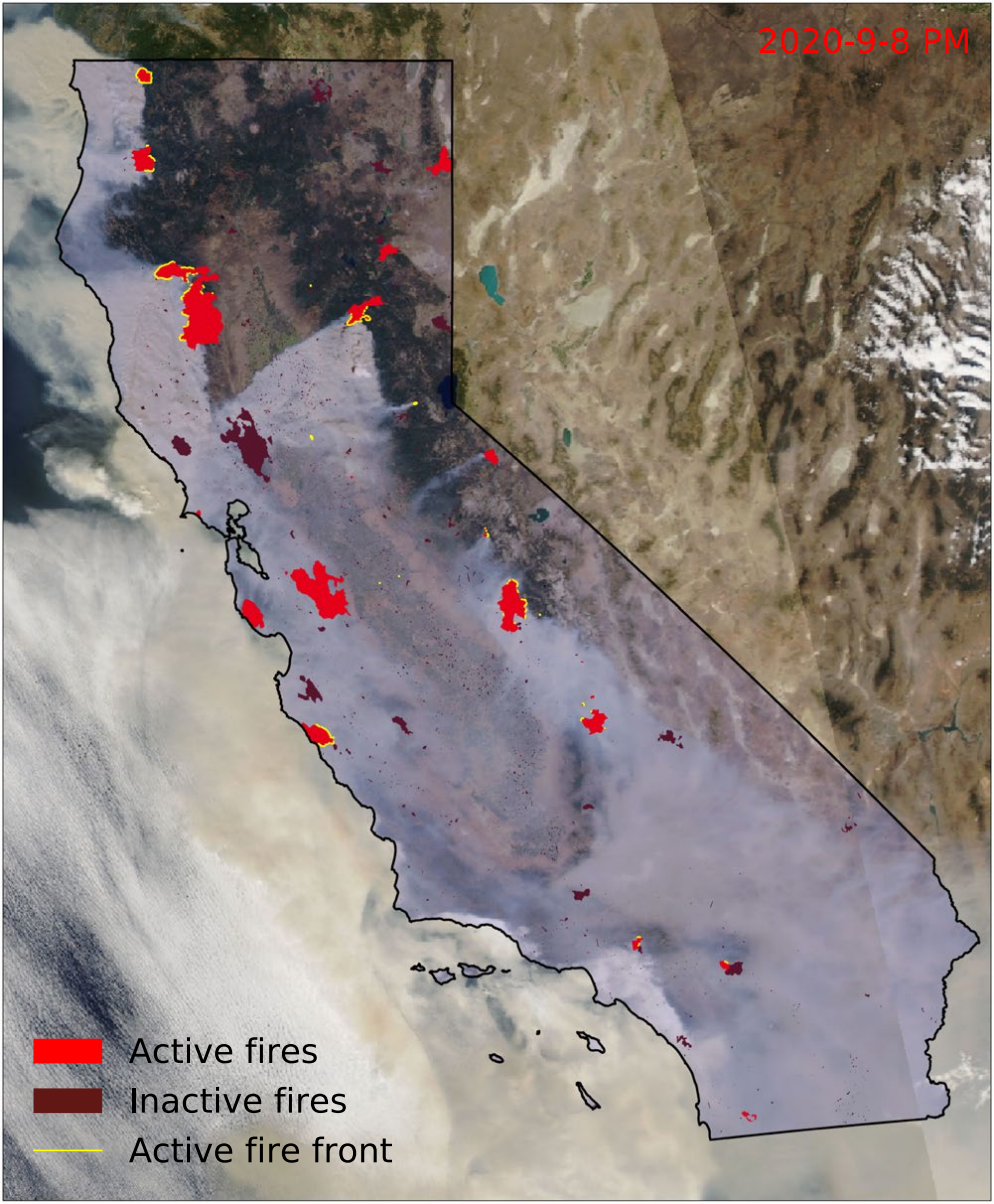
An example map of fire perimeters and fire active fronts in California. The map was created using the fire event data suite (FEDS) as of the Suomi-NPP afternoon overpass (~1:30 pm local time) of Sep 8, 2020. The background is the Aqua MODIS Corrected Reflectance Imagery (true color) recorded at the same day (provided by the NASA Global Imagery Browse Services). The active front line of a fire is shown in yellow, active fire areas are shown in red, and the area of inactive (extinguished) fires are shown in dark red.

We also calculate the active front line for each *fire* object at each time step. The active fire front consists of the segments of the fire perimeter that are actively burning and releasing energy and emissions. The position of the active fire line is critical in evaluating the fire risk, estimating the fire emissions, and predicting fire spread. We derive the active portion of the fire perimeter as segments that are within a 500 m radius of newly detected fire pixel locations. We found that this threshold allowed for a continuous projection of the active fire front in rapidly expanding areas of large wildfires during the 2018 fire season; this threshold may be optimized in future work to maximize performance metrics for fire model forecasts. The resulting active line for each fire at each time step has the shape of a linestring (object representing a sequence of points and the line segments connecting them), a multi-linestring (a collection of multiple linestrings), or a linear ring (closed linestring). Figure 5 shows an example map of the fire perimeters and active fire front lines on September 8 during the 2020 wildfire season.

Additional fire properties, such as the fire area and active fire line length, are also derived using these geometries of the *fire* object (see Table 2). Note this list can be easily expanded to include more user-defined properties with the help of the *fire* object core vector data.

The *allfires* object contains a list of all existing *fire* objects at a time step. This object also records the *id*s of fire objects that have been modified (including fires newly formed, fires that expanded with new pixel additions, fires with pixels addition due to merging, and fires that just became invalid) at the current time step.

### Creating the fire event data suite (FEDS)

By tracking the spatiotemporal evolution of all *fire* objects in California, we derived a complete dataset of fire events for each calendar year (Jan 1 am – Dec 31 pm) during the Suomi-NPP VIIRS era (2012–2020). The dataset contains four products that represent the fire information in California at multiple spatial scales and from different perspectives (Fig. 1 and Table 4), ranging from the most detailed and memory-intensive data format (Pickle) to the most high-level format (CSV).

**Table 4:** Data structure of the FEDS.

PRODUCT NAME	PURPOSE	FILES		
		Name	Format	Contents
**Serialization**	Full access to all attributes associated with all levels of fire objects at each time step	**YYYYMMDDAP**.pkl	Pickle	Serialization of the fire object
**Snapshot**	Easy access to major attributes associated with each *fire* object at each time step	**YYYYMMDDAP**.gpkg	Gpkg	Fire attributes and perimeter
		Finalperimeter_2012-2020.gpkg	Gpkg	Final fire perimeters and attributes for all fires during 2012–2020
**Largefire**	Easy access to time series of major attributes associated with large fire objects	LargeFires_**YYYY**.gpkg	Gpkg	Time series of large fire attributes and perimeter
		LargeFires_2012–2020.gpkg	Gpkg	Time series of attributes and perimeter for all large fires during 2012–2020
**Summary**	Summary of regional statistics of all fire events within a year	fsummary_**YYYY**1231PM.nc	NetCDF	Time series of regional fire statistics
		Flist_heritage_**YYYY**.csv	CSV	List of fire merging history
		Flist_large_**YYYY**.csv	CSV	List of large fire *id*s

In the file name, ‘YYYY’ indicates the four-digit year number, ‘MM’ indicates the two-digit month number, ‘DD’ indicates the two-digit day number, ‘AP’ indicates the two-letter string of morning (‘AM’) or afternoon (‘PM’) overpasses. In each GeoPackage (.gpkg) file, there are three data layers, with ‘perimeter’ layer storing fire attributes and perimeters, ‘fireline’ layer storing fire active front lines, and ‘newfirepix’ layer storing positions of newly detected fire pixels.

The first product is the direct serialization result of the *allfires* object at each time step (twice per day). The product is stored as a Pickle file^31^ which allows for analysis of the complex *allfires* object structure (including all attributes associated with all *fire* objects it contains). This file also serves as the restart file for continued fire tracking at any time step, which is essential for the operational mode using the near-real-time fire data. By restoring an exact copy of the previously pickled *allfires* object, any attribute in the *allfires* object can be deserialized from the saved files. The Pickle file is the most basic data product in the dataset, and is created at each half-day time step.

The second product (Snapshot) represents a more accessible and self-explanatory variant of the Pickle serialization product. In this product, we tabulated important diagnostic attributes for each fire and saved them in GeoPackage^32^ data files. Each GeoPackage file includes three data layers: one contains the properties and the fire perimeter geometry, another contains the active fire line geometry, and a third contains the new fire pixel location geometry. This product, created at a half-daily time step, allows for a more straightforward interpretation of regional fire status at a particular time step. We also created a GeoPackage file that summarizes the final fire perimeters and attributes for all fires during the whole study period (2012–2020).

**Fig. 6:**
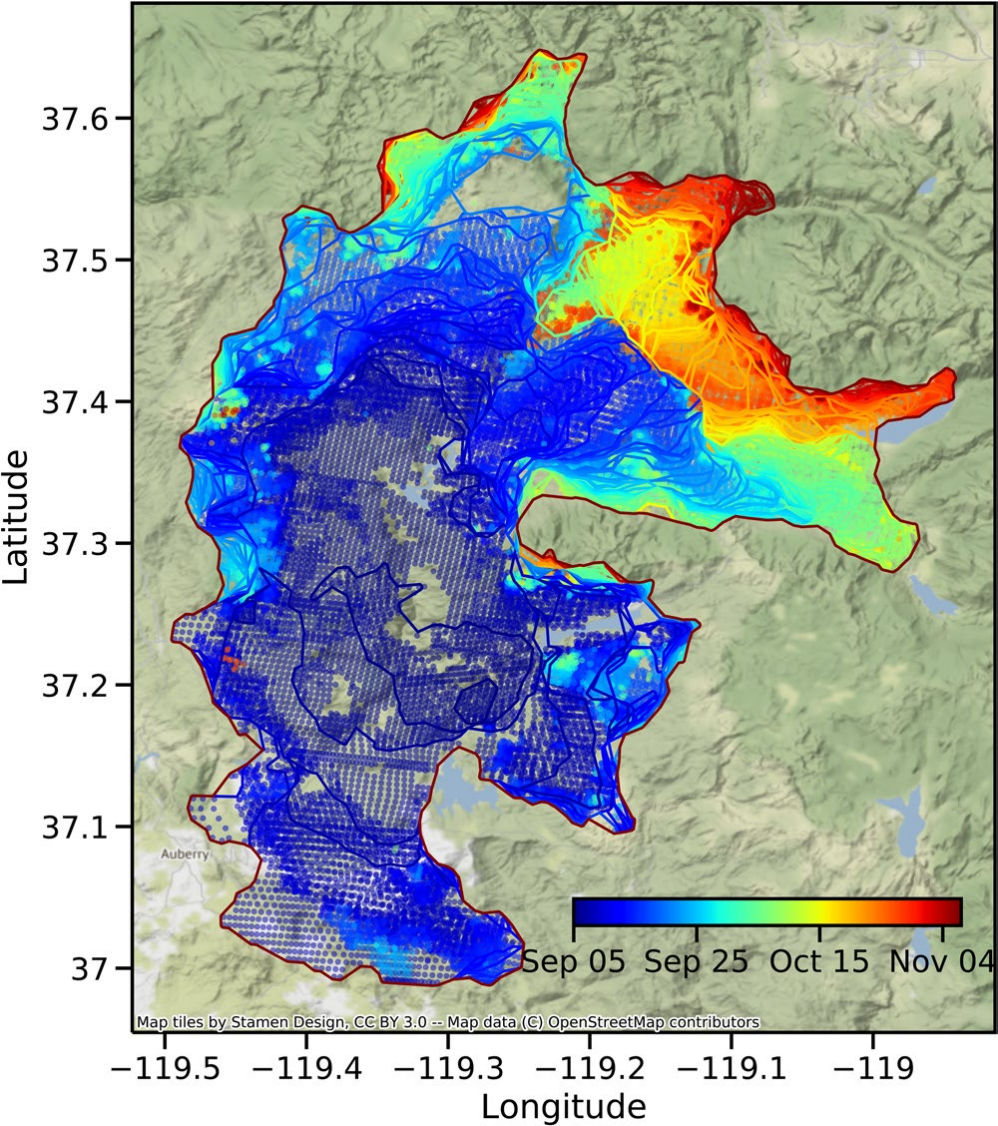
The spatiotemporal evolution of the Creek fire (2020). Contours and dots reflect the fire perimeters and newly detected fire pixels at each 12-hour time step. Data for the period of Sep 5 am–Nov 6 am, 2020 are shown.

The third product (Largefire) focuses on the temporal evolution of individual large fires with an area greater than 4 km^2^. At each time step, the time series of properties and geometries (fire perimeter, active fire line, and new fire pixel locations) for each of the large fires are extracted and saved to GeoPackage files. This product facilitates the visualization and analysis for an individual targeted fire (Fig. 6) and is particularly useful in the near-real-time evaluation, forecasting, and policy making.

The fourth product (Summary), which is stored as NetCDF and CSV files and created at the end of a fire season, records the all-year time series of fire statistics (including major fire attributes such as number, size, duration, fire line length, etc.) over the whole State of California. This product provides a feasible regional summary of the temporal evolution of fires.

### Potential for near-real-time (NRT) fire event tracking

While the main objective of this paper is to apply the object-based fire tracking system to historical VIIRS fire detections and create a retrospective multi-year FEDS, we note that this system has the potential to be used for tracking fire events in near-real-time, providing rich and valuable information for fire management and short-term risk assessment. We have experimented with the use of this system for NRT fire event tracking in California using the daily NRT Suomi-NPP VIIRS active fire detection product (VNP14IMGTDL, collection 6) as the main data source. The VNP14IMGTDL product is routinely produced and is publicly available at the NASA Fire Information for Resource Management System (FIRMS). Since the NRT product undergoes less rigorous quality assurance, we use only fires with ‘*nominal’* or ‘*high’* confidence levels from the NRT product for fire tracking. Some active fire detections from the NRT data are potentially associated with static non-vegetation fires (*e.g*., fires from gas flaring in oil and gas or landfill industries or false detections due to reflection from solar panels) and are not the main interest for vegetation fire studies. To avoid the unnecessary computation associated with these static fires, we record and evaluate the fire pixel density for each *fire* object at each time step. When a small fire (<20 km^2^) has an exceptionally persistent fire signal (*i.e*., with cumulative fire pixel density since ignition > 20 per km^2^), it is considered to be a static fire and subsequently labelled as *invalid*.

Similar to the retrospective FEDS, we use the active fire detections to create an object serialization product, a regional snapshot GIS product, and a time series product of large fire evolution twice daily. This experimental NRT data will be available upon publication through a university hosted server.

## Data Records

The FEDS data records for the 2012–2020 fire seasons can be accessed via the *figshare* data repository^33^.

The dataset provides four compressed files for each year (Table 4): (i) *Serialization.tar.gz* contains all Pickle (.pkl) files that store half-daily *allfires* objects; (ii) *Snapshot.tar.gz* contains GeoPackage (.gpkg) files that store major fire attributes and geometries at half-daily time step, as well as a GeoPackage file that store the final geometry and attributes of all fires; (iii) *Largefire.tar.gz* contains GeoPackage files of large fire time series, and (iv) *Summary.tar.gz* include several year-end summary files in the formats of NetCDF and comma separated values (CSV). We recorded a total of 35337 active fire objects in California over the 2012–2020 period. The GeoPackage file of large fire time series includes 735 fires with a final size greater than 4 km^2^, and 12801 records of 12-hour growth increments.

## Technical Validation

### Comparison of the final fire perimeters with FRAP

The Fire and Resource Assessment Program (FRAP), established by the California Department of Forestry and Fire Protection, develops the fire perimeter GIS layer for public and private lands throughout California at the end of each calendar year (http://frap.fire.ca.gov/frap-projects/fire-perimeters). The FRAP fire perimeter database is widely viewed as the most complete digital record of fire perimeters in California. While the main objective of this study is to track the spread dynamics of individual fires and not to map the burned area precisely, a comparison of our year-end fire objects for 2018 to the FRAP database does provide a partial validation of the ability of our algorithm to accurately classify the size and shape of large wildfires. Note that the fires from FRAP and our fire objects do not always have a one-to-one match. The FEDS data recorded a much greater number of small fires. Sometimes, a single FRAP fire can correspond to multiple fire objects in FEDS. In the comparison, we used all fire objects that had spatial overlap with each FRAP fire.

**Fig. 7:**
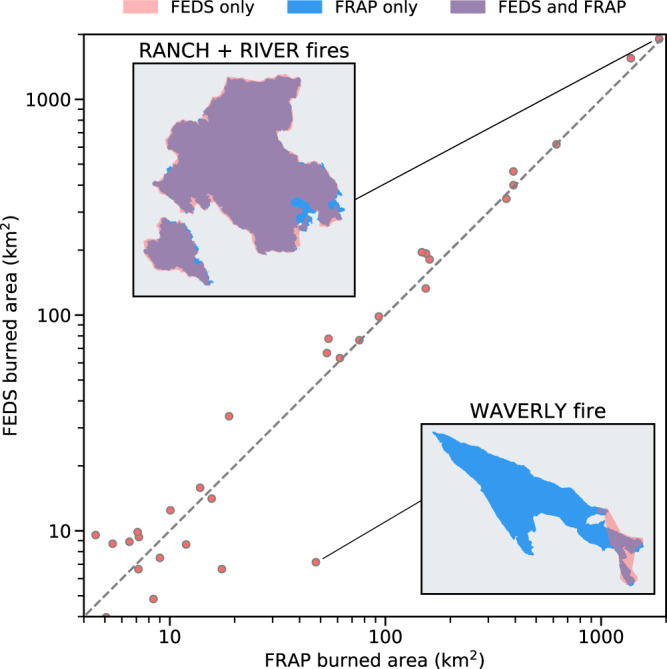
Comparison of fire perimeter final sizes from the FEDS with FRAP burned area. Each dot represents a large fire (final size > 4 km^2^) occurring in California during 2018. Purple areas in inset figures show regions of agreement for the two example wildfires. FEDS fire perimeter generally agrees well with FRAP, but sometimes underestimates the burned area for fast moving grassland fires, such as the Waverly Fire.

**Fig. 8:**
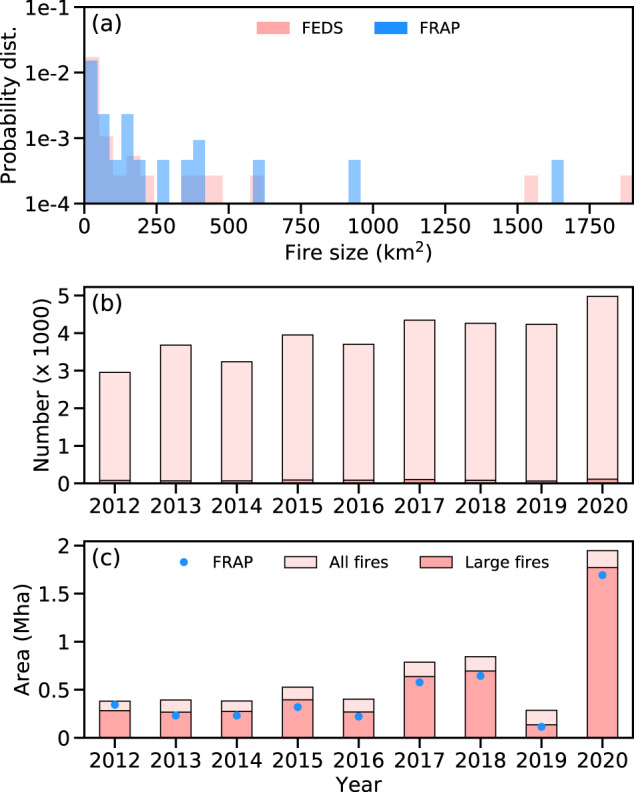
Comparison of fires from FEDS and FRAP. (**a**) The fire size distributions are calculated from all large fires occurring in California during 2018. (**b**) Interannual variability of fire object numbers from the FEDS dataset over California during 2012–2020. (**c**) Similar to (**b**), but for fire burned areas. Large fires are fires with a final size greater than 4 km^2^.

Overall, the final fire perimeters for 2018 from the California FEDS agreed well with that from the FRAP dataset (Tables 5 and 6). The total burned areas from the two sources were similar (with a ratio of 1.091 over California), and the slope (Fig. 7) and accuracy (Table 6) were very close to 1. The spatial overlap of burned areas (two examples are shown in Fig. 4) was close, and all performance metrics (precision, recall, intersection over union, F1 score) varied between 0.71 and 0.93 on a per fire basis and between 0.79 and 0.93 on a regional basis (Table 6). Commission errors were partly due to the inclusion of unburned islands as a result of the vector perimeter algorithm. It is also important to note that the FRAP dataset does not capture all small fires, and this also contributes to the mismatch between two datasets. The agreement in size distributions of all large fires (Fig. 8a) suggests that the fire object approach does not have a systematic bias in grouping active fire pixels. The inter-annual variability in the total area within the fire perimeters from this study also agreed well with the FRAP data (Fig. 8b,c).

**Table 5:** Confusion matrix of the comparison between FEDS year-end fire perimeters and FRAP burned area in the State of California.

	FEDS: UNBURNED	FEDS: BURNED	SUM
**FRAP: UNBURNED**	(TN) 40.65	(FP) 0.11	(FRAP_UB = TN + FP) 40.76
**FRAP: BURNED**	(FN) 0.05	(TP) 0.61	(FRAP_B = FN + TP) 0.66
**SUM**	(FEDS_UB = TN + FN) 40.70	(FEDS_B = FP + TP) 0.71	(AREA_TOTAL) 41.42

Areas of comparison (in Mha) are derived using all fires occurring in California during 2018. The FRAP data are considered as the actual class and the FEDS data are considered as the predicted class. TN: True Negative; FN: False Negative; FP: False Positive; TP: True Positive; FEDS_UB: Unburned area from FEDS; FEDS_B: Area burned from FEDS; FRAP_UB: Unburned area from FRAP; FRAP_B: Burned area from FRAP; AREA_TOTAL: Total land area in California.

**Table 6:** Scores of FEDS fire perimeter by comparing with FRAP or NIFC data using all fires occurring in California during 2018.

METRICS	DEFINITION	FRAP		NIFC	
		REGIONAL	PER FIRE	REGIONAL	PER FIRE
**Ratio**	FEDS_B/REF_B	1.091	1.145 ± 0.403	1.198	1.298 ± 0.409
**Accuracy**	(TP+TN)/AREA_TOTAL	0.996	—	—	—
**Precision**	TP/FEDS_B	0.847	0.827 ± 0.145	0.788	0.744 ± 0.157
**Recall**	TP/REF_B	0.925	0.923 ± 0.252	0.944	0.943 ± 0.166
**Intersection Over Union (IOU)**	TP/(TP + FP + FN)	0.794	0.706 ± 0.212	0.753	0.725 ± 0.149
**F1 score**	2 * (Precision * Recall)/(Precision + Recall)	0.884	0.847 ± 0.192	0.859	0.850 ± 0.079

**Fig. 9:**
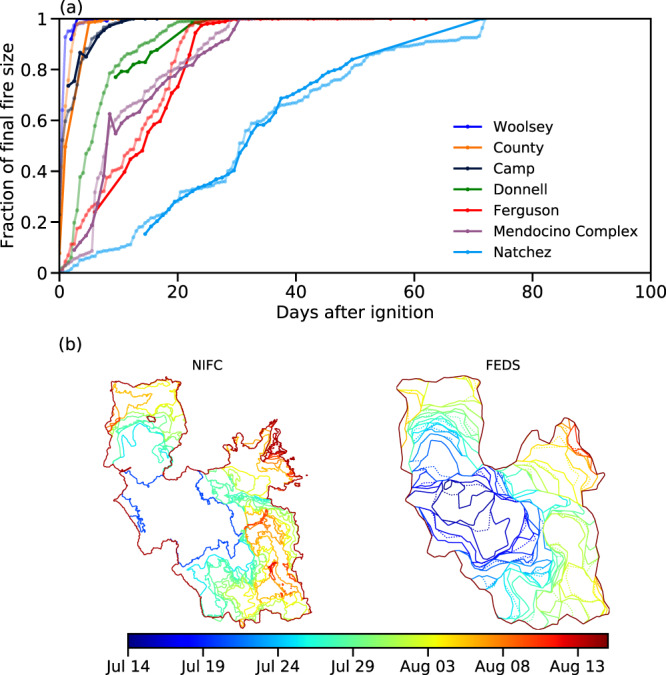
Comparison of the temporal progression of large fires from FEDS with that from NIFC. (**a**) Time series of burning fraction (representing the size of a fire object normalized by the final sizes from each dataset) at each time step after ignition. FEDS data are shown in light-coloured lines and NIFC data in dark-coloured lines. Among the large fires occurring in California during 2018, seven fires were selected for comparison based on the availability of NIFC data, fire duration, and the one-to-one correspondence between two datasets (Sometimes a single fire object from FEDS is spatially overlapped with multiple fires from NIFC). With this plot, the fires are ordered from left to right from shortest duration to longest duration. Note that the NIFC daily fire perimeter data were only available for part of the fire duration. (**b**) Spatial progression of Ferguson fire (2018) as defined from FEDS and NIFC. For FEDS, the ‘AM’ fire perimeters are shown in solid lines and the ‘PM’ fire perimeters are shown in dotted lines.

For comparison to FRAP data, only the year-end fire perimeters are used. The REGIONAL columns represent the agreement in the whole region of California. The PER FIRE columns represent the mean values and 1-σ uncertainty for comparisons with each individual large fire in the FRAP or NIFC dataset. In the NIFC column, we list the scores by comparing all valid fire perimeters of large fires shown in Fig. 9a with the afternoon (‘PM’) fire perimeters from FEDS for each day. Accuracy is only reported for comparisons with FRAP in the whole region of California (FRAP REGIONAL column). Area values in the DEFINITION column are calculated from the confusion matrix as defined in Table 5. REF_B represents area burned from FRAP or NIFC.

### Comparison of fire progressing with the NIFC event polygons

The National Interagency Fire Center (NIFC) Incident Feature Service provides daily polygons for some historical fire incidents in the US using multiple sources of observational data. We extracted daily fire polygons for 7 large fires occurring in California during the 2018 fire season (https://data-nifc.opendata.arcgis.com/maps/nifc::national-incident-feature-service-2018) and compared the temporal progressing of the fire area with that derived in our fire object-based FEDS dataset. We found the daily evolution of the fire size (Fig. 9a) and spatial range (Fig. 9b) for these fires from the FEDS dataset agreed reasonably well with the NIFC data. For the set of 7 fires in the NIFC dataset (representing 262 daily snapshots), performance metrics (precision, recall, intersection over union, F1 score) varied between 0.72 and 0.95 on a per fire basis, and between 0.75 and 0.95 across the full set of fires (Table 6).

### Data limitations and uncertainty

The dataset’s quality is influenced by both the uncertainty in VIIRS active fire detections and limitations of the algorithm we used for dynamical tracking of wildland fire perimeters.

The VIIRS I-band fire product has a relatively high spatial resolution (375 m at nadir) and therefore has a more minor omission error than its predecessors (such as MODIS). But the pixel size for the VIIRS fire product is much larger at the edge of the image swath due to oblique scanning, which increases the uncertainty in the sub-pixel fire location. This may also reduce the detection efficiency, particularly for small or cool fires. Larger pixel areas at the edge of a scan make it more challenging to determine the actual area occupied by a VIIRS fire pixel. In the current algorithm, we assume the fire is located at the centre of each fire-affected pixel.

Thermal anomalies reported in the VIIRS fire location data do not always represent real fire detections. Occasionally, smoke plumes within the lower atmosphere can be hot enough to be classified as fire pixels. Due to the parallax effect, the surface geo-registration of these pixels can have a spatial discrepancy relative to the location of the actual fire burning on the surface, leading to incorrect determination of fire perimeter and active fire front line. Fire aerosols and clouds also make it difficult to detect active fires.

Fires can spread rapidly and go undetected during the time between repeat satellite overpasses. This is particularly an issue for grassland fires in windy conditions (*e.g*., the Waverly fire shown in Fig. 7) because low fuel loads limit the time an area will support flaming-phase combustion - which is more easily observed by the VIIRS sensor. Our algorithm assumes all areas between two consecutive fire perimeters are burned during that time step. This method reduces omission errors which are common in the active fire-based approaches, but also may misclassify unburned islands, leading to an overestimation of the burned area. Compared to previous studies, this issue is partly improved in our approach by using fire detections with a higher spatial resolution (VIIRS I-band data) and recording the fire perimeters at a higher temporal resolution (12-hour intervals). We compared the interior area (with a 1-km buffer) of the final perimeters from FEDS with MODIS burned area product (MCD64A1) for all large fires occurred in California during 2018, and estimated that 9% of area within the fire perimeter may be unburned islands. This is comparable to the unburned fraction (12%) derived from the FRAP fire perimeter^34^.

The accuracy of the fire identification and fire perimeters is not only limited by the quality of satellite fire detections but also depends on the connectivity parameters used for grouping pixels and fire objects, as well as the alpha shape parameter used to estimate the fire perimeter. We use land cover dependent connectivity parameters and optimize the alpha shape parameter (see the Methods section) in this study to improve the accuracy. Together, considering the spatial resolution of VIIRS observations (with a mean pixel size of ~470 m across the swath width), the potential for a wildfire to occupy only a small area within a VIIRS pixel (see section ‘Methods’ above), and additional uncertainties introduced from the use of an optimized alpha-shape parameter (Fig. 4), we estimate the mean uncertainty related to the spatial position of any specific perimeter location is approximately ± 500 m (defined as ± 1 standard deviation). Errors may be larger in rare instances where hot smoke plumes (instead of surface fires) trigger the fire detection algorithm, yielding a projection of active fire locations onto land surfaces that may be outside the true fire perimeter.

### Data completeness and scalability

Suomi-NPP was launched on October 2011, and the VIIRS active fire data are available since Jan 20, 2012. We applied the algorithm presented here to all fire season days and created the Californian fire event database for 2012–2020. As mentioned before, the same method can also be used to create a near-real-time database for each 12-hour time step using the VIIRS NRT fire product which NASA regularly updates. In the future, our algorithm may allow for tracking fires at a higher temporal resolution if additional active fire observations become available with comparable spatial resolution and geolocation accuracy at complementary satellite overpass times.

With the rich information associated with each fire, including the fire perimeter and active fire front, an important next step is to extend this dataset by deriving other fire attributes by combining it with other data sources (e.g., land surface and meteorology).

Since the VIIRS active fire data is a global product, the system presented in this paper can be scaled up to quantify fire behaviour and spread rates in other regions with minor modifications. The fire tracking approach in the system can also be applied to other sources of active fire data. Specifically, integration of additional information from other VIIRS sensors on NOAA 20 and JPSS 2 (planned launch in Sept 2022) will provide additional capability to resolve fire perimeters by providing multiple view angles and by increasing the likelihood of measuring surface conditions (and active fires) in areas with extensive smoke and cloud cover.

## Usage Notes

We provide example Python scripts (in SampleCode.py) to read the four types of products that comprise this dataset (Table 5).

The fire attributes at a time step (half-daily) can be read from the GeoPackage files using the *read_gpkg* function. The function returns a Geopandas DataFrame (GDF), with the index column representing the fire indices, geometry column representing vector shapes of the fire perimeter, active fire line, and the new active fire locations, and other columns representing other fire properties (if available).

Similarly, the time series of attributes for large fires stored in GeoPackage files can also be loaded into GDFs. For each large fire, the time column of the GDF spans the whole period starting from the ignition time and fire ending time (the last time step with active fire detection).

The year-end statistical summary is a self-explanatory NetCDF file and can be read into memory using the *read_netcdf* function. The time dimension spans the whole half-daily time steps (from Jan 1st am to Dec 31th pm) for each year. The list of fire merging history (heritage) and large fires can be read from file using the *read_csv* function.

The *allfires* object stored in the Pickle serialization files can be read using the *read_pickle* function. Properties related to the *allfires* object, as listed in Table 3, are available for extraction and evaluation. All available *fire* objects, including those associated with invalid fires, are included in the list variable *allfires.fires*. The indices in the list represent fire identification numbers (*fids*). The attributes associated with individual fires can be extracted using a specific fire *id* – *allfires.fires[fid]*. Similarly, the properties of individual active fire *pixel* object can be evaluated using *allfires.fires[fid].pixels[pid]*, in which *pid* is the unique pixel *id* within a fire.

## Data Availability

The open-source Python code of the fire tracking system, as well as sample scripts for reading the dataset, are freely available at the *figshare* data repository^33^, along with the 2012–2020 FEDS dataset. Versions and packages of the Python script include *Numpy* (1.17.5), *Pandas* (1.0.1), *Geopandas* (0.7.0), *Xarray* (0.15.0), *Scipy* (1.4.1), *Shapely* (1.7.1), *Gdal* (3.0.4), and *Pyproj* (2.5.0).
